# Sex differences in iron status during military training: a prospective cohort study of longitudinal changes and associations with endurance performance and musculoskeletal outcomes

**DOI:** 10.1017/S0007114523001812

**Published:** 2024-02-28

**Authors:** Thomas J. O’Leary, Sarah Jackson, Rachel M. Izard, Neil P. Walsh, Charlotte V. Coombs, Alexander T. Carswell, Samuel J. Oliver, Jonathan C. Y. Tang, William D. Fraser, Julie P. Greeves

**Affiliations:** 1 Army Health and Performance Research, Army Headquarters, Andover, MA, UK; 2 Division of Surgery and Interventional Science, UCL, London, UK; 3 Defence Science and Technology, Ministry of Defence, Porton Down, Porton, UK; 4 Faculty of Science, Liverpool John Moores University, Liverpool, UK; 5 Norwich Medical School, University of East Anglia, Norwich, UK; 6 School of Health Sciences, University of East Anglia, Norwich, UK; 7 College of Human Sciences, Bangor University, Bangor, UK; 8 Norfolk and Norwich University Hospital, Norwich, UK

**Keywords:** Bone, Endurance, Musculoskeletal Injury, Nutrition

## Abstract

This study investigated sex differences in Fe status, and associations between Fe status and endurance and musculoskeletal outcomes, in military training. In total, 2277 British Army trainees (581 women) participated. Fe markers and endurance performance (2·4 km run) were measured at the start (week 1) and end (week 13) of training. Whole-body areal body mineral density (aBMD) and markers of bone metabolism were measured at week 1. Injuries during training were recorded. Training decreased Hb in men and women (mean change (–0·1 (95 % CI –0·2, –0·0) and –0·7 (95 % CI –0·9, –0·6) g/dl, both *P* < 0·001) but more so in women (*P* < 0·001). Ferritin decreased in men and women (–27 (95 % CI –28, –23) and –5 (95 % CI –8, –1) µg/l, both *P* ≤ 0·001) but more so in men (*P* < 0·001). Soluble transferrin receptor increased in men and women (2·9 (95 % CI 2·3, 3·6) and 3·8 (95 % CI 2·7, 4·9) nmol/l, both *P* < 0·001), with no difference between sexes (*P* = 0·872). Erythrocyte distribution width increased in men (0·3 (95 % CI 0·2, 0·4)%, *P* < 0·001) but not in women (0·1 (95 % CI –0·1, 0·2)%, *P* = 0·956). Mean corpuscular volume decreased in men (–1·5 (95 % CI –1·8, –1·1) fL, *P* < 0·001) but not in women (0·4 (95 % CI –0·4, 1·3) fL, *P* = 0·087). Lower ferritin was associated with slower 2·4 km run time (*P* = 0·018), sustaining a lower limb overuse injury (*P* = 0·048), lower aBMD (*P* = 0·021) and higher beta C-telopeptide cross-links of type 1 collagen and procollagen type 1 N-terminal propeptide (both *P* < 0·001) controlling for sex. Improving Fe stores before training may protect Hb in women and improve endurance and protect against injury.

Fe is a trace element that contributes to normal physiological function by incorporation into enzymes – including those involved in energetic metabolic pathways – and proteins involved in oxygen transport – including Hb (> 65 % total body Fe)^(1)^. Fe is also a mineral component of bone and contributes to the synthesis of collagen^(2,3)^, a significant component of musculoskeletal tissues. Fe status is determined by the measurement of a combination of blood biochemical markers including ferritin, transferrin saturation, soluble transferrin receptor (sTfR), erythrocyte distribution width (RDW), mean corpuscular volume (MCV) and Hb^(4–7)^. Fe deficiency is defined as low Fe stores before Hb levels are affected and Fe deficiency anaemia is defined as low Fe stores and low Hb^(1,6)^. There are several criteria for defining Fe deficiency (low Fe stores), which complicates our interpretation of Fe deficiency and its effect on health and performance. The WHO defines depleted Fe stores as ferritin < 15 μg/dl for men and women, and anaemia as Hb < 12 g/dl for women and < 13 g/dl for men^(7)^. Suboptimal Fe status leads to poorer endurance and cognitive performance, lethargy and fatigue and impaired aerobic adaptations to training^(6)^ and may contribute to poorer musculoskeletal outcomes including poorer vitamin D status^(2)^, lower bone mineral density^(2,3)^ and increased injury risk^(8,9)^.

There are well-described differences in Fe status between men and women; more women than men have Fe deficiency and Fe deficiency anaemia in the general^(7,10)^ and military^(11)^ populations. Premenopausal women are at high risk of Fe deficiency due to menstrual blood loss and insufficient dietary Fe intake to meet these increased Fe requirements from menstrual bleeding^(6,12)^. Therefore, poor Fe status may play a more important role in the health and performance of women than men, particularly in environments where Fe intake and Fe stores are challenged^(3)^. Basic military training diminishes Fe status in women^(5,13–15)^ with better Fe status associated with better endurance performance^(13,14)^. Similar observations have been made in men^(16–18)^, but there are little data comparing the effect of military training in men and women. A study of US Army basic training found military training degraded Fe status to a greater extent in women compared with men^(19)^, but these data were on a relative small sample and no study has studied sex differences in the effect of training on Fe status in a UK Armed Forces population or examined the association between Fe status and measures of musculoskeletal health. Women are now fully integrated into all roles in the UK and US Armed Forces and operate alongside men in the most arduous roles. Understanding sex differences in Fe status is essential in optimising health and performance of both sexes. Women experience higher physical demands^(20)^ and more musculoskeletal injuries^(21)^ and consume less Fe despite higher daily requirements^(22)^ than men in military training. Fe status in male and female British Army recruits and its association with endurance performance and musculoskeletal outcomes will provide important insight into methods to protect health and performance.

The primary aim of this study was to examine sex differences in changes in Fe status during British Army basic military training. The secondary aims were to explore associations between markers of Fe status and endurance performance, musculoskeletal injury incidence, areal bone mineral density, vitamin D status and biochemical markers of bone metabolism. We hypothesised that Fe status deteriorates during military training to a greater extent in women than men. We also hypothesised that markers of Fe status would be associated with endurance performance and musculoskeletal outcomes.

## Methods

### Participants

The study was advertised to new British Army trainees from April 2013 to July 2017 during week one of their basic training courses. Participants were recruited from three British Army training populations: male infantry recruits at Infantry Training Centre, Catterick; standard entry (non-infantry and non-officer) female recruits at Army Training Centre, Pirbright and male and female officer cadets at Royal Military Academy, Sandhurst, providing a representative sample of all individuals commencing British Army basic training. All participants passed an initial military medical assessment and were confirmed to be injury free and not have any medical condition that precluded military service. This study was conducted according to the guidelines laid down in the Declaration of Helsinki, and all procedures were approved by the Ministry of Defence Research Ethics Committee (ref: 165/Gen/10). Each participant had the study procedures and risks fully explained verbally and in writing. Written informed consent was obtained from all participants.

### Experimental design

This study was an observational prospective cohort study. These data were secondary analyses as part of a larger study exploring micronutrient deficiencies and health and performance outcomes^(23–25)^. Venous blood samples were drawn at the start (week 1) and end (week 13) of each basic military training course for the analysis of biochemical markers of Fe status, vitamin D status and biochemical markers of bone metabolism. Endurance performance was assessed by a maximal effort 2·4 km run at the same time points. Body mass, height and whole-body areal bone mineral density measured by dual-energy X-ray absorptiometry were recorded at week 1. Participants self-reported their alcohol intake, use of Fe supplementation, smoking habits and stress fracture history using questionnaires at week 1. Week 1 measurements were made following the initial medical assessment and before military training commenced. Male infantry recruits completed the 26-week British Army infantry basic training course or the 28-week British Army parachute regiment course. Standard entry female recruits completed the 14-week British Army soldier basic military training course. The officer cadets completed the 44-week officer commissioning course. The first 14 weeks of each British Army basic military training course are similar between training sites and intended to develop basic military skills and physical fitness. The 14-week training programme involves periods of aerobic endurance training, strength and conditioning, military-specific fitness training (obstacle course, circuit training), military drill, progressive loaded marching and basic military skills (field exercise, weapon handling). Week 14 involves a decrease in typical military activities and an increase in the administrative burden as trainees prepare to complete the basic training component of their course; post-training measurements were, therefore, taken in week 13. Participants’ medical records were accessed to obtain a record of clinician-diagnosed lower limb overuse injuries and lower limb stress fractures (including hip/pelvis) during the first 14 weeks of training; lower limb stress fractures were recorded separately from lower limb overuse injuries.

### Blood collection and biochemical analyses

A venous blood sample was collected either in the morning (∼09.00 to 11.00 h) after breakfast (06.00 to 07.00 h) or early afternoon (∼13.00 to 15.00 h) after lunch (12.00 to 13.00 h). Follow-up measurements were taken at approximately the same time of day. Venous blood was withdrawn from a vein in the antecubital fossa and collected in serum and EDTA BD Vacutainer® tubes (Becton Dickinson). Serum samples were left to clot for 1 h at room temperature. Hb, RDW and MCV were measured in EDTA whole blood within 30 min of collection using the COULTER A^C^ · T diff 2 Analyzer (Beckman Coulter). Blood samples were centrifuged at 1500 *g* and 4°C for 10 min before serum and plasma were separated into universal tubes and stored at –80°C until analysis. Plasma procollagen type 1 N-terminal propeptide (PINP), beta C-telopeptide cross-links of type 1 collagen (*β*CTX), intact parathyroid hormone (PTH) and serum ferritin were analysed by electro-chemiluminescence immunoassays on the COBAS c601 (Roche Diagnostics) platform. PINP inter-assay CV was < 3 % between 20 and 600 µg/l with a sensitivity of 8 µg/l. *β*CTX inter-assay CV was < 3 % between 200 and 150 µg/l with a sensitivity of 10 ng/l. PTH inter-assay CV was < 3·8 % between 1·2 and 5000·0 pg/ml. Ferritin inter-assay CV was < 4·2 % between 0·5 and 2000·0 µg/l. Serum sTfR was measured by immunoturbidimetric assays performed on the COBAS c501 analyser (Roche Diagnostics). sTfR inter-assay CV was < 6·0 % between 5·9 and 472·0 nmol/l. Serum samples were analysed for total 25-hydroxyvitamin-D (25(OH)D) by liquid chromatography tandem mass spectrometry^(26)^. The 25(OH)D3 and 25(OH)D2 assays were calibrated using the National Institute of Science and Technology standard reference material SRM972a. Total 25(OH)D was calculated from the sum of 25(OH)D3 and 25(OH)D2. Total 25(OH)D inter-assay CV was < 8·5 % between 0·1 and 200·0 nmol/l. All biochemical analyses (excluding Hb, RDW and MCV analyses) were undertaken by the Good Clinical Laboratory Practice and Vitamin D External Quality Assessment Scheme (DEQAS)-certified Bioanalytical Facility at the University of East Anglia, Norwich, UK.

### Endurance performance

Endurance performance was assessed as the time to complete a maximal effort 2·4 km run on a standardised running course at each training site. Participants completed an 800 m warm-up and completion time was recorded to the nearest second. The time to complete a 2·4 km run is indicative of maximal aerobic capacity^(27)^ and is a military field test assessed during selection, training and throughout a military career. All participants were accustomed to performing this test from selection, before commencing military training.

### Whole-body areal bone mineral density

Whole-body areal bone mineral density was assessed by dual-energy X-ray absorptiometry (Lunar iDXA, GE Healthcare), with men wearing underwear and women wearing light clothing. Scans were not performed on men and women at the Royal Military Academy, Sandhurst due to lack of scanner availability at that site.

### Statistical analyses

These data were secondary analyses^(23–25)^ and so no a priori sample size was calculated for Fe markers. The lowest number of follow-up measurements was for RDW for women (120 observations). Based on a two group sample size of 240, the smallest effect size we could detect was a partial eta-squared (η_p_^2^) of 0·01 with a power of 80 % and *α* of 0·05. All data were analysed using the R programming language (*v.*4.2.2). Distribution of the demographic and anthropometric data was checked using frequency distribution histograms. Participant demographics and anthropometrics at week 1 were compared between men and women with independent samples *t* tests or a Welch’s *t* test for groups with unequal variances. The number of men and women using Fe supplements was compared using a *χ* ^2^ test. Linear mixed effect models with restricted maximum likelihood estimation were used to compare changes in markers of Fe status (Hb, ferritin, sTfR, RDW and MCV) and 2·4 km run time in men and women during training (*lme4 package v*.1.1.31). Sex (men *v*. women), time (week 1 *v*. week 13) and their interaction were included as fixed effects to examine sex differences. Random intercepts were assigned to each participant to account for within-participant correlation for repeated measures. Significance of the fixed effects from each model was determined with Satterthwaite df (*lmerTest package v*.3.1.3). Variance and normality of the residuals for each model were checked visually by plotting the residuals against the fitted values and from Q-Q plots. Data were log transformed for models where the residuals seriously violated these assumptions. *P* values were corrected with the Holm–Bonferroni method (*n* 5 *P* values for five Fe status outcomes). In the event of a significant interaction, pairwise comparisons with Holm–Bonferroni corrections and Kenward–Roger df were used on the linear mixed effects model to identify differences between time points within each sex and differences between each sex within each time point (*emmeans package v*.1.8.3). Pooled data were used for main effects when there was no significant interaction. Effect sizes are presented as η_p_^2^ for main and interaction effects, Cohen’s d for between-group comparisons and paired Cohen’s d for within-group paired comparisons (*effectsize package v*.0.8.2).

Simple linear regression was used to test the associations between each marker of Fe status and 2·4 km run time at both week 1 and week 13 in men and women separately. *P* values were corrected with the Holm–Bonferroni method (*n* 5 *P* values for five Fe status outcomes at each time point). Multiple linear regression was used to test the association between each marker of Fe status and 2·4 km run time at week 1 controlling for age, sex, BMI, smoking and alcohol intake^(23)^. Multiple linear regression was used to test the association between absolute change in each marker of Fe status with absolute change in run time controlling for that respective Fe marker and run time at week 1. Binary logistic regression was performed to assess the association between injury (lower limb overuse injury or lower limb stress fracture) and each marker of Fe status controlling for sex, BMI, 2·4 km run time, total 25(OH)D, smoking, stress fracture history and Army training course (infantry, standard entry or officer). Army training type was included to account for differences in training site, equipment and other practices that may contribute to differences in musculoskeletal injury incidence. Multiple linear regression was used to test the association between each marker of Fe status and musculoskeletal outcomes (whole-body areal bone mineral density, total 25(OH)D, PTH, *β*CTX and PINP) controlling for sex and BMI. Each marker of Fe status was entered separately into each multiple linear regression and binary logistic regression models (five models per outcome). Variance and normality of the residuals for simple and multiple linear models were checked visually by plotting the residuals against the fitted values and from Q-Q plots. Data were log transformed for models where the residuals seriously violated these assumptions. Figures were drawn in the *ggplot2 package* (*v.*3.4.0). Significance was accepted as *P* ≤ 0·05.

## Results

### Participants

**Fig. 1.**
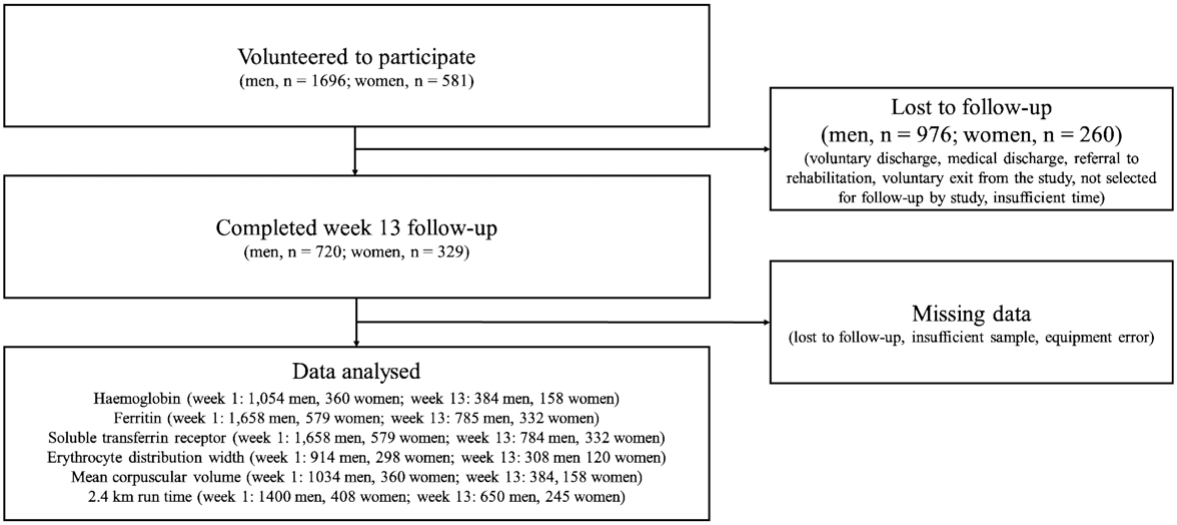
Participant flow through the study.

In total, 2277 British Army trainees (1696 men and 581 women, Table 1) volunteered to participate. Men were heavier (*P* < 0·001) and taller (*P* < 0·001) than women, but age was not different between sexes (*P* = 0·933); more women than men took a Fe supplement (*P* = 0·023) (Table 1). A total of 1049 (720 men and 329 women) completed week 13 testing (Fig. 1).

**Table 1.** Participant demographics and anthropometrics (Numbers and percentages; mean values and standard deviations)

	Men (*n* 1696)		Women (*n* 581)	
	*n*	%	*n*	%
Training type				
Infantry	1293	76	0	0
Standard entry	0	0	475	82
Officer	403	24	106	18
	Mean	sd	Mean	sd
Age (years)	22	8	22	3
Body mass (kg)	76·2	9·9	64·8	7·9*
Height (m)	1·78	0·06	1·65	0·06*
BMI (kg·m^2^)	24·1	2·6	23·7	2·3
Taking Fe supplements				
*n*	62		30	
%	3·8 %		5·4 %*	

Missing data: age, 41 men and 4 women; body mass, 46 men and 14 women; height, 46 men and 12 women; BMI, 46 men and 16 women; taking Fe supplements, 61 men and 29 women.

* *P* < 0·05 *v*. men.

### The effect of training on Fe status

**Fig. 2.**
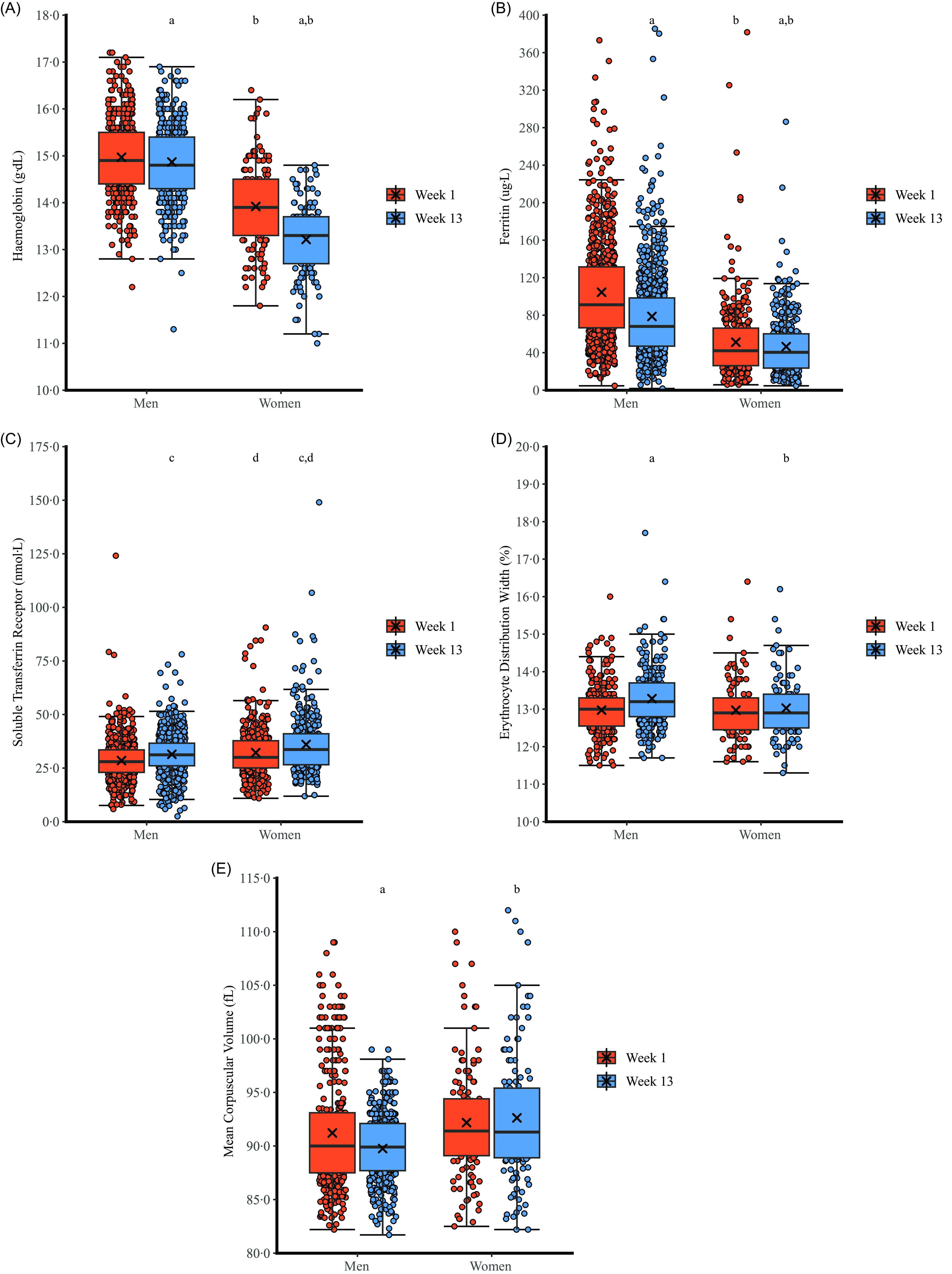
The effect of military training on markers of Fe status in men and women. Box plots represent median, interquartile range and range. Crosses represent mean. ^a^ *P* < 0·05 *v*. week 1 (within sex); ^b^ *P* < 0·05 *v*. men at same time-point; ^c^ *P* < 0·05 *v*. week 1 (main effect of training); ^d^ *P* < 0·05 *v*. men (main effect of sex). Data are truncated at 80·0 fL for mean corpuscular volume for clarity; three men had a value < 80·0 fL at week 1 and week 13

Biochemical markers of Fe status are presented in Fig. 2 with mean absolute changes presented in Table 2. Examination of the residuals showed that ferritin, sTfR and MCV errors had heteroscedasticity and long-tailed distributions and so results are reported for log transformed data. There was a sex × time interaction for Hb (*P* < 0·001, η_p_^2^ = 0·094). Post-hoc pairwise comparisons revealed that Hb decreased in men (*P* < 0·001, d_z_ = 0·16) and women (*P* < 0·001, d_z_ = 0·76) with the decrease greater in women. Hb was higher in men than women at week 1 (*P* < 0·001, d = 1·25) and week 13 (*P* < 0·001, d = 2·06). There was a sex × time interaction for log ferritin (*P* < 0·001, η_p_^2^ = 0·042). Post-hoc pairwise comparisons revealed that log ferritin decreased in men (*P* < 0·001, d_z_ = 0·74) and women (*P* = 0·001, d_z_ = 0·15) with a greater decrease in men. Log ferritin was higher in men than women at week 1 (*P* < 0·001, d = 1·33) and week 13 (*P* < 0·001, d = 0·96). There was no sex × time interaction for log sTfR (*P* = 0·873, η_p_^2^ < 0·001), but training increased log sTfR (main effect of time, *P* < 0·001, η_p_^2^ = 0·062) and log sTfR was higher in women than men (main effect of sex, *P* < 0·001, η_p_^2^ = 0·035). There was a sex × time interaction for RDW (*P* < 0·001, η_p_^2^ = 0·026). Post-hoc pairwise comparisons revealed that RDW increased in men (*P* < 0·001, d_z_ = 0·48) but not in women (*P* = 0·956, d_z_ = 0·08). RDW was not different between men and women at week 1 (*P* = 0·194, d = 0·09) but was higher in men than women at week 13 (*P* = 0·003, d = 0·31). There was a sex × time interaction for log MCV (*P* < 0·001, η_p_^2^ = 0·031). Post-hoc pairwise comparisons revealed that log MCV decreased in men (*P* < 0·001, d_z_ = 0·40) but not in women (*P* = 0·087, d_z_ = 0·08). Log MCV was not different between men and women at week 1 (*P* < 0·001, d = 0·12) but was lower in men than women at week 13 (*P* < 0·001, d = 0·52).

**Table 2.** Mean absolute change in markers of Fe status and 2·4 km run time from week 1 to week 13 of military training in men and women (95 % confidence intervals)

	Men		Women	
	Mean absolute change	95 % CI	Mean absolute change	95 % CI
Hb (g/dl^1^)	–0·1	–0·2, –0·0	–0·7	–0·9, –0·6
Ferritin (µg/l^1^)	–27	–28, –23	–5	–8, –1
Soluble transferrin receptor (nmol/l^1^)	2·9	2·3, 3·6	3·8	2·7, 4·9
Erythrocyte distribution width (%)	0·3	0·2, 0·4	0·1	–0·1, 0·2
Mean corpuscular volume (fL)	–1·5	–1·8, –1·1	0·4	–0·4, 1·3
2·4 km run time (s)	–31	–34, –39	–27	–31, –22

### The effect of training on endurance performance

**Fig. 3.**
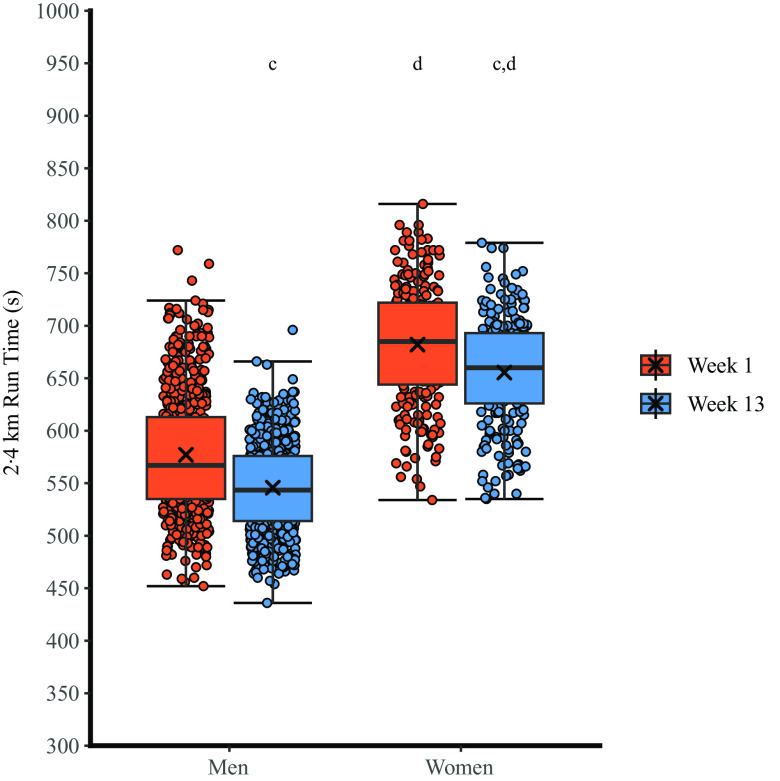
The effect of military training on 2·4 km run time in men and women. Box plots represent median, interquartile range and range. Crosses represent mean. ^a^ *P* < 0·05 *v*. pre-training (within sex); ^b^ *P* < 0·05 *v*. men at same time-point; ^c^ *P* < 0·05 *v*. pre-training (main effect of training); ^d^ *P* < 0·05 *v*. men (main effect of sex).

There was no sex × time interaction for 2·4 km run time (*P* = 0·125, η_p_^2^ = 0·003), but training decreased 2·4 km run time (main effect of time, *P* < 0·001, η_p_^2^ = 0·371) and 2·4 km run time was faster in men than women (main effect of sex, *P* < 0·001, η_p_^2^ = 0·431) (Fig. 3, Table 2).

### Associations between Fe status and endurance performance

**Fig. 4.**
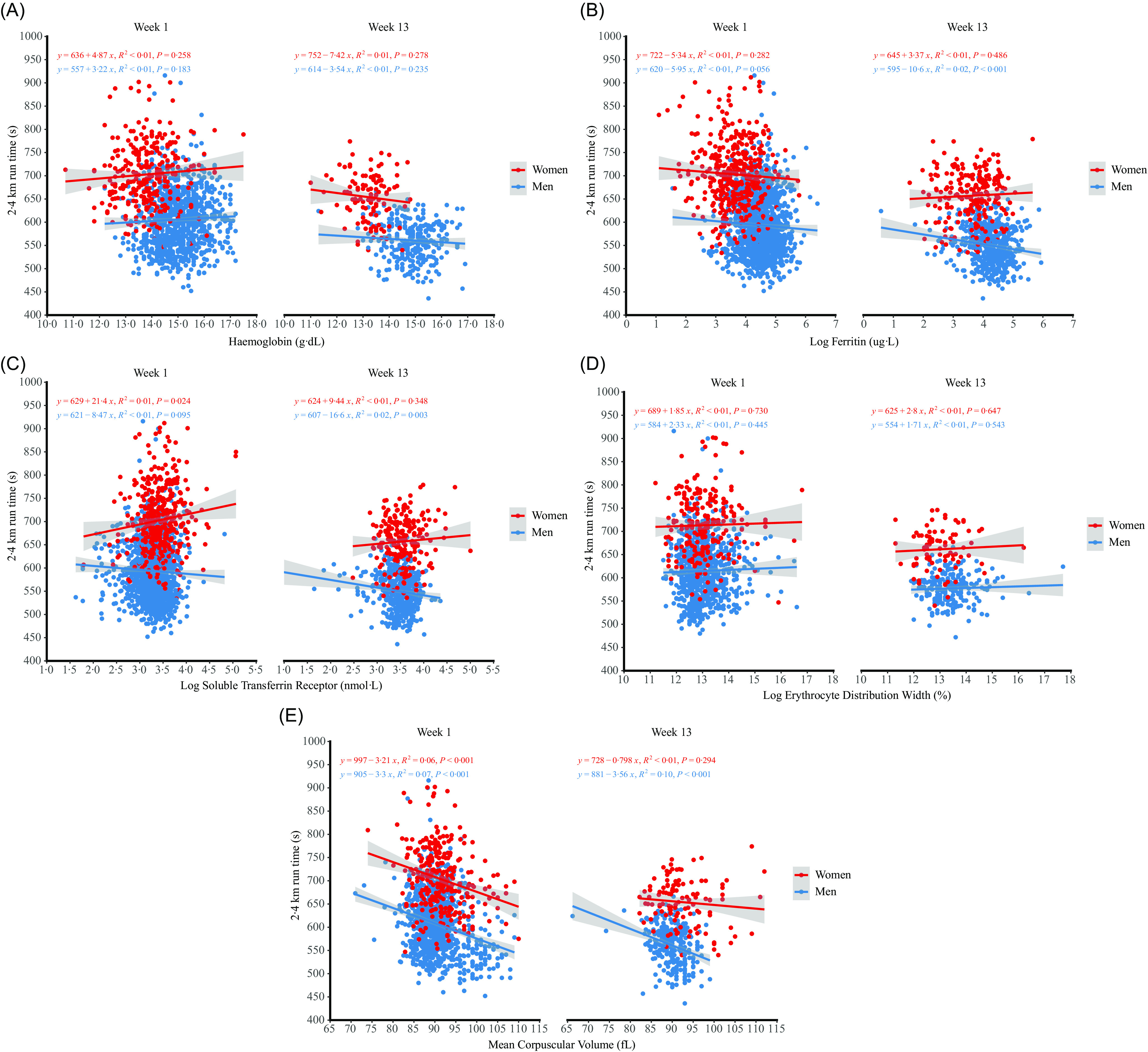
Relationships between markers of Fe status and 2·4 km run time at week 1 and week 13 of military training in men and women. *P* values are unadjusted for multiple comparisons. Adjusted *P* values are presented within the text.

Simple linear regressions between markers of Fe status and 2·4 km run time are presented in Fig. 4. Examination of the residuals showed that ferritin and sTfR errors had heteroscedasticity and long-tailed distributions and so simple linear regression results are reported for log transformed data. There was no evidence of associations between Hb or RDW and 2·4 km run time at either time-point in men or women (*r* ≤ 0·07, p ≥ 0·470). There was a significant negative relationship between log ferritin and 2·4 km run time at week 13 in men only (*r* = –0·15 (95 % CI –0·24, –0·07), *P* < 0·001) but no evidence of an association between log ferritin and 2·4 km run time at week 1 in or at either time-point in women (*r* ≤ 0·05, *P* ≥ 0·224). There was a significant negative association between log sTfR receptor and 2·4 km run time at week 13 in men only (*r* = –0·13 (95 % CI –0·21, –0·05), *P* = 0·009) but no evidence of an association between log sTfR and 2·4 km run time at week 1 in men or at either time-point in women (*r* ≤ 0·11, *P* ≥ 0·096). There were negative associations between MCV and 2·4 km run time in men at week 1 (*r* = –0·26 (95 % CI –0·32, –0·20), *P* < 0·001) and week 13 (*r* = –0·31 (95 % CI –0·41, –0·20), *P* < 0·001) and in women at week 1 (*r* = –0·25 (95 % CI –0·36, –0·14), *P* < 0·001) but not week 13 (*r* = –0·10 (95 % CI –0·28, 0·08), *P* = 0·976). There was a negative association between ferritin and MCV with 2·4 km run time at week 1 and a positive association between log sTfR and 2·4 km run time at week 1, when controlling for age, sex, BMI, smoking and alcohol intake; there was no evidence of association between Hb or RDW with 2·4 km run time at week 1 (Table 3). There were positive associations between change in ferritin, sTfR and MCV with change in 2·4 km run time when controlling for sex, concentrations of the respective marker at week 1 and 2·4 km run time at week 1; we found no evidence of an association between change in Hb and change in RDW and change in 2·4 km run time (Table 4).

**Table 3.** Association between markers of Fe status and 2·4 km run time (s) at week 1 (95 % confidence intervals)

Marker of Fe status*	Coefficient	95 % CI	*P*
Hb (g/dl)	1·78	–2·53, 5·89	0·433
Ferritin (µg/l)	–0·07	–0·13, –0·01	0·018
Soluble transferrin receptor (nmol/l)	0·33	0·02, 0·64	0·036
Erythrocyte distribution width (%)	0·79	–4·35, 5·94	0·762
Mean corpuscular volume (fL)	–3·21	–3·94, –2·47	< 0·001

* Controlling for age, sex, BMI, smoking status and alcohol intake.

**Table 4.** Association between changes in markers of Fe status and changes in 2·4 km run time (s) from week 1 to week 13 (95 % confidence intervals)

Marker of Fe status*	Coefficient	95 % CI	*P*
Change in Hb (g/dl)	–4·38	–8·77, 0·02	0·051
Change in ferritin (µg/l)	0·10	0·03, 0·16	0·006
Change in soluble transferrin receptor (nmol/l)	0·23	0·02, 0·43	0·030
Change in erythrocyte distribution width (%)	3·60	–1·65, 8·84	0·178
Change in mean corpuscular volume (fL)	1·20	0·40, 2·00	0·003

* Controlling for sex, week 1 2·4 km run time and week 1 marker of Fe status.

### Associations between Fe status and musculoskeletal injury incidence

Associations between markers of Fe status and injury incidence controlling for sex, BMI, 2·4 km run time, total 25(OH)D, smoking status, previous stress fracture and training course can be seen in Table 5. The incidence of at least one lower limb overuse injury was 24·1 % for men and 34·0 % for women (26·5 % for both sexes combined) and for lower limb stress fractures was 2·9 % for men and 2·5 % for women (2·9 % for both sexes combined). There was no evidence of associations between Hb, sTfR, RDW or MCV at week 1 and developing a lower limb overuse injury or a stress fracture. Lower ferritin at week 1 was associated with developing a lower limb overuse injury, but there was no evidence of ferritin being associated with developing a lower limb stress fracture.

**Table 5.** Associations between markers of Fe status at week 1 and injury incidence (Odds ratios and 95 % confidence intervals)

	Lower limb overuse injury			Lower limb stress fracture		
Marker of Fe status*	OR	95 % CI	*P*	OR	95 % CI	*P*
Hb (g/dl)	1·055	0·894, 1·246	0·524	1·114	0·741, 1·674	0·603
Ferritin (µg/l)	0·998	0·995, 1·000	0·048	0·999	0·992, 1·004	0·667
Soluble transferrin receptor (nmol/l)	1·008	0·997, 1·019	0·143	1·005	0·977, 1·027	0·662
Erythrocyte distribution width (%)	1·074	0·877, 1·310	0·486	0·915	0·544, 1·457	0·723
Mean corpuscular volume (fL)	0·988	0·951, 1·027	0·545	1·046	0·944, 1·158	0·390

* Controlling for sex, BMI, 2·4 km run time, total 25(OH)D, smoking status, previous stress fracture and training course.

### Association between Fe status and bone mineral density and markers of bone metabolism

Associations between markers of Fe status and areal bone mineral density and markers of bone metabolism controlling for sex and BMI can be seen in Table 6. There was no evidence of Hb or RDW being associated with areal bone mineral density or markers of bone metabolism at week 1. Higher ferritin was associated with higher areal bone mineral density and lower *β*CTX and PINP. Higher sTfR was associated with higher PINP and higher PTH. Higher MCV was associated with higher total 25(OH)D and lower PTH, *β*CTX and PINP.

**Table 6.** Associations between markers of Fe status and bone mineral density and markers of bone metabolism at week 1 (95 % confidence intervals)

	Whole-body aBMD (g/cm)			Total 25(OH)D (nmol/l)			PTH (pg/ml)			*β*CTX (μg/l)			PINP (μg/l)		
Marker of Fe status*	Coefficient	95 % CI	*P*	Coefficient	95 % CI	*P*	Coefficient	95 % CI	*P*	Coefficient	95 % CI	*P*	Coefficient	95 % CI	*P*
Hb (g/dl)	–0·003	–0·010, 0·003	0·289	–0·085	–1.723, 1·553	0·919	0·010	–0·060, 0·080	0·774	0·002	–0·010, 0·013	0·783	–0·740	–3.078, 1·599	0·535
Ferritin (µg/l)	0·000	0·000, 0·000†	0·021	–0·023	–0·047, 0·001	0·059	–0·001	–0·002, 0·000	0·077	0·000	–0·001, 0·000‡	< 0·001	–0·152	–0·182, –0·123	< 0·001
Soluble transferrin receptor (nmol/l)	0·000	0·000, 0·001	0·540	0·105	–0·013, 0·224	0·081	0·013	0·008, 0·018	< 0·001	0·000	–0·001, 0·001	0·830	0·166	0·017, 0·316	0·029
Erythrocyte distribution width (%)	–0·007	–0·014, 0·001	0·097	1·706	–0·374, 3·786	0·108	0·043	–0·044, 0·129	0·332	–0·001	–0·015, 0·013	0·888	1·289	–1·741, 4·319	0·404
Mean corpuscular volume (fL)	0·000	–0·002, 0·001	0·773	0·595	0·300, 0·891	< 0·001	–0·021	–0·033, –0·008	0·002	–0·006	–0·008, –0·004	< 0·001	–1·250	–1·671, –0·825	< 0·001

aBMD, areal bone mineral density; *β*CTX, beta C-telopeptide cross-links of type 1 collagen; PINP, procollagen I N-terminal propeptide; PTH, parathyroid hormone; total 25(OH)D, total 25-hydroxyvitamin D.

* Controlling for sex and BMI.

† coefficient = 0·0003.

‡ coefficient = –0·0005.

## Discussion

Training resulted in widespread and sex-specific changes in markers of Fe status, indicative of poorer Fe status at the end of training in both men and women. Lower Fe stores (lower ferritin) were associated with poorer endurance performance, higher musculoskeletal injury incidence, lower whole-body areal bone mineral density and higher biochemical markers of bone metabolism. Therefore, these data have important implications for managing the health and performance of men and women in the British Army. The large sample size also provides novel insight into sex differences in Fe metabolism and associations with musculoskeletal outcomes in young adults.

### The effect of training on Fe status

Training resulted in widespread disturbances to Fe status; Hb and ferritin decreased, and sTfR increased in men and women. Training also increased RDW and decreased MCV – red blood cell size characteristics – but only in men. These changes in Fe markers are all indicative of poorer Fe status at the end of military training^(13)^. Hb is the most abundant of the Fe containing (heme-) proteins and is vital for oxygen transport^(1)^. Ferritin reflects Fe stores in the liver, spleen and bone marrow^(1)^ but can be impacted by inflammation, acute phase response and pathologies, although very few conditions other than Fe deficiency decrease ferritin^(28)^. Previous studies of women in basic military training have shown training decreased ferritin and increased sTfR, similar to our data, but increased Hb and RDW^(5,13–15)^. Conversely, we observed a decrease in Hb in both sexes and an increase in RDW in men only. There is evidence of decreased Hb in men and mixed samples of men and women after basic military training courses^(16–18)^, supporting our findings. The data from our study and these previous studies show military training results in poorer Fe status, consistent with other data showing poorer Fe status in athletes compared with non-athletes^(29,30)^. There are fewer military studies directly comparing men and women, but US Army basic military training decreased ferritin and increased sTfR more in women than men^(19)^; our study builds on these findings by exploring sex differences with a larger sample size and by providing the first data in a UK population undergoing a different military training programme. Differences between our study findings and these previous studies could be due to differences in demographics of participants, training length, training modalities and sample size.

Training decreased Hb more in women but decreased ferritin more in men. A decrease in Hb could be due to plasma volume expansion, but the changes in other markers of Fe status support the mechanism is depletion of Fe^(13)^. Complete depletion of Fe stores can occur before Hb is decreased, with low Hb a late phase of Fe deficiency^(1)^. Women had lower Fe stores (lower ferritin) than men on entry to military training. These lower Fe stores could explain why women were more susceptible to developing decreases in Hb, whereas men experienced greater decreases in Fe stores (decreases in ferritin), protecting Hb. Decreased MCV in men supports evidence of impaired erythropoiesis, but this decrease in cell size appears due to attenuated macrocytosis (MCV > 100 fL) rather than the development of microcytosis (MCV < 80 fL) with all men with macrocytosis at week 1 in the normal range by week 13. These sex differences could be due to differences in Fe status before training rather than any sex difference in response to training. However, several factors may increase the risk of developing Fe deficiencies in basic military training for women. The physical demands of military training are typically higher for women than men^(20)^ and so exercise-induced Fe losses could be greater for women. Basic military training may increase Fe losses through gastrointestinal bleeding, sweat loss, haematuria, haemolysis from ground impact forces and eccentric muscle contraction and increased inflammation and hepcidin^(5,6,13,29–31)^. Hepcidin is a key regulator of Fe status and inhibits Fe absorption^(31)^ with the increase in hepcidin with exercise dependent on initial Fe status and therefore potentially sex^(29)^. There is also evidence that women consume less Fe than men in basic military training despite higher daily requirements^(19,22,32)^. Menstrual bleeding is a primary cause of Fe loss in women^(12)^, although the effect of basic military training on menstrual blood loss is not clear^(33)^ and a large proportion of Servicewomen take hormonal contraceptives that stop menstrual bleeding^(34)^. Finally, sex differences in circulating sex steroid concentrations may contribute to sex differences in Fe status; sex steroids play a role in erythropoiesis and may influence Fe metabolism, with the female sex steroids potentially influencing hepcidin^(29)^.

### Associations between Fe status and endurance performance

Higher ferritin concentrations, higher MCV and lower sTfR were associated with a faster 2·4 km run time at week 1 in multiple linear regressions controlling for sex and other factors known to influence endurance performance. The 2·4 km run is used as a test of aerobic fitness on entry and in-service in the British Army and is related to maximal oxygen uptake^(27)^, although other factors such as submaximal running economy likely contribute to performance. Low ferritin and high sTfR are early signs of Fe deficiency and these data show that better Fe stores are associated with better endurance performance, although the confidence intervals around the coefficients were wide and so the exact estimates are not clear. Visual examination of the sTfR data by sex and week of training (Fig. 4) suggests that the sTfR and 2·4 km run time are sex dependent, although this interaction was not formally tested. Fe deficiency and Fe deficiency anaemia have different physiological effects with implications for performance; tissue oxidative capacity is impaired with Fe deficiency with oxygen-carrying capacity reduced with Fe deficiency anaemia^(6,35)^. We observed no relationship between Hb and run time, but few people had low Hb so performance may not be impacted at the Hb concentrations observed in our study. Higher levels of tissue Fe and increased activity of Fe-dependent oxidative enzymes could have contributed to better endurance performance^(35)^, although other mechanisms like less subjective lethargy may contribute^(29)^. Higher sTfR was associated with slower 2·4 km run time^(14)^, and higher RDW and lower Hb were associated with slower 3·2 km run time^(13)^ in basic military training in other nations. These studies did not find evidence of an association between run time and ferritin, but they did not control for other factors known to influence performance. Laboratory studies show ferritin is positively associated with maximal oxygen uptake independently of Hb in women^(36)^. Fe supplementation increases ferritin and maximal oxygen uptake in those with low ferritin and normal Hb^(37)^, but not all studies show endurance performance improvements when ferritin is increased^(31,38)^. Fe supplementation improved 3·2 km run time in female military recruits, but only those with Fe deficiency anaemia^(5)^, with the efficacy of Fe supplementation on improving performance likely dependent on starting Fe status^(39)^.

The largest improvements in 2·4 km run time were associated with the largest decreases in ferritin, sTfR and MCV. There is some evidence that better Fe status contributes to better training adaptations^(6)^, but these data show greater degradation of Fe stores contributes to better aerobic training adaptations. A possible explanation is that those experiencing the highest training load improved performance the most and also had the largest decreases in ferritin. The decrease in MCV was largely due to attenuated macrocytosis (men with MCV > 100 fL, Fig. 2(e)) rather than microcytosis and it is not clear if attenuated macrocytosis is beneficial for performance. These data are in contrast to a previous military study showing bigger increases in sTfR change were associated with poorer improvements in 3·2 km run time in women during basic military training^(13)^. The magnitude of the association between change in markers of Fe status and change in endurance performance is not clear due to the wide confidence intervals around the coefficients.

### Associations between Fe status and musculoskeletal outcomes

Lower ferritin at the start of training was associated with developing a lower limb overuse injury during training, but the effect was small and there was no evidence of an association between Fe status and developing a lower limb stress fracture. Previous military studies have shown no difference in Fe status between men who develop a stress fracture and those who do not^(18)^, but there is evidence of an increased prevalence of Fe deficiencies in women with stress fractures than controls^(8,9)^. Low aerobic capacity is a risk factor for musculoskeletal injuries^(40)^ and was associated with Fe status; however, we controlled for 2·4 km run time in our analyses and so better fitness is unlikely to explain why better Fe stores were associated with lower injury incidence. Fe is a mineral component of bone and contributes to the synthesis of collagen^(2,3)^, a significant component of musculoskeletal tissues. Therefore, Fe deficiency could contribute to musculoskeletal injuries through the degradation of musculoskeletal tissues. We observed a positive association between ferritin and whole-body areal bone mineral density – in agreement with studies in animals^(41)^ and dietary intake studies in postmenopausal women^(42)^ – supporting a role for Fe status in musculoskeletal health. Lower ferritin and MCV were associated with higher rates of bone turnover – higher *β*CTX and PINP – which may contribute to the lower whole-body areal bone mineral density. Similar associations between Fe markers and bone metabolism have been shown previously^(43)^, and treatment of Fe deficiency with Fe supplements decreased markers of bone resorption and formation^(44)^. Lower areal bone mineral density and higher markers of bone metabolism with lower ferritin could be due to the effects of reduced Fe availability on enzyme activity involved in collagen synthesis, and altered osteoblasts and osteoclasts activity due to reduced prolyl hydroxylase activity and altered cell signalling^(2)^. Higher sTfR was associated with higher PTH and higher MCV was associated with higher total 25(OH)D and lower PTH, so the mechanism could be through the effects of Fe deficiency on the PTH-1-*α*-hydroxylase axis^(2)^.

### Limitations

This study did not measure circulating concentrations of hepcidin or transferrin, which could have helped further explain some of our findings. Men and women also completed different training courses; however, the first 14 weeks of all basic military training courses are programmed to be similar. We did not control for plasma volume in our analysis of Hb; however, our other markers of Fe status support a decline in Fe status as a mechanism for decreased Hb. We did not control for physical activity before the blood sample and it is not clear if acute changes in inflammation influenced our results. The length of our study was also only several months and longer periods of study may be required to detect changes in Fe status and for the clinical effects of Fe depletion to manifest. Finally, we did not measure Fe intake or menstrual bleeding or menstrual status during training, which may explain the changes we observed in women.

### Conclusions

British Army basic training resulted in widespread disturbances to Fe status, indicative of poorer Fe status at the end of training in both men and women. Men had a larger decrease in Fe stores (early-stage Fe deficiency), whereas women had a larger decrease in Hb (late-stage Fe deficiency). Better Fe stores were associated with better endurance performance, lower incidence risk of musculoskeletal injury, higher bone mineral density and lower circulating concentrations of markers of bone metabolism. Increasing Fe intake should be a consideration before and during military training to protect health and performance for men and women but is particularly important for women to reduce the risk of developing Fe deficiency anaemia^(3)^.
